# Domain-specific perceptual causality in children depends on the spatio-temporal configuration, not motion onset

**DOI:** 10.3389/fpsyg.2013.00365

**Published:** 2013-07-11

**Authors:** Anne Schlottmann, Katy Cole, Rhianna Watts, Marina White

**Affiliations:** Developmental Science Department, University College LondonLondon, UK

**Keywords:** perception of causality, social causality, physical causality, causal reasoning, domain-specificity, agency, animacy, cognitive development

## Abstract

Humans, even babies, perceive causality when one shape moves briefly and linearly after another. Motion timing is crucial in this and causal impressions disappear with short delays between motions. However, the role of temporal information is more complex: it is both a cue to causality and a factor that constrains processing. It affects ability to distinguish causality from non-causality, and social from mechanical causality. Here we study both issues with 3- to 7-year-olds and adults who saw two computer-animated squares and chose if a picture of mechanical, social or non-causality fit each event best. Prior work fit with the standard view that early in development, the distinction between the social and physical domains depends mainly on whether or not the agents make contact, and that this reflects concern with domain-specific motion onset, in particular, whether the motion is self-initiated or not. The present experiments challenge both parts of this position. In Experiments 1 and 2, we showed that not just spatial, but also animacy and temporal information affect how children distinguish between physical and social causality. In Experiments 3 and 4 we showed that children do not seem to use spatio-temporal information in perceptual causality to make inferences about self- or other-initiated motion onset. Overall, spatial contact may be developmentally primary in domain-specific perceptual causality in that it is processed easily and is dominant over competing cues, but it is not the only cue used early on and it is not used to infer motion onset. Instead, domain-specific causal impressions may be automatic reactions to specific perceptual configurations, with a complex role for temporal information.

## Introduction

Humans, including infants from 6 months, perceive causality when one geometric shape moves briefly after another, on a linear path. Motion timing is crucial in this and causal impressions disappear with short delays between the motions. However, the role of temporal information in perceptual causality is more complex than this: it provides not only cues to causality but is also a processing factor. It affects not only ability to distinguish causality from non-causality, as commonly emphasized, but also to distinguish social from mechanical causality. Here we consider this wider role of temporal information with children aged 3 to 7 years and adults.

Perceptual causality in motion sequences obviously lacking real causality has been the topic of much research since Michotte's (1963) seminal work, which continues to attract interest because it promises one simple solution to the complex problem of how we know about cause and effect. Michotte argued that in some cases we do not need to know, but can simple see causality. Rather than requiring much experience with relevant events, and complex reasoning to link the experienced events to another, causality appears as a Gestalt property of particular motion sequences. Just as we see a triangle when shown three appropriately configured corners (Kanizsa, 1976), we see causality, one event producing another, when shown two motions in appropriately configured sequence. This provides us with a perceptual identification of what cause is that does not require any conceptual knowledge or understanding.

**Figure 1:**
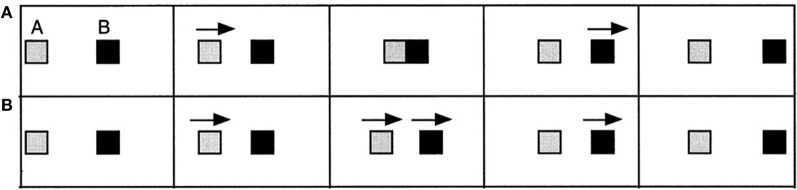
**Michotte's (1963) launch event (top—A) and Kanizsa and Vicario's reaction event (bottom—B)**.

Michotte's prime example was the launch event, in which shape A moved up to and contacted a stationary shape B and stopped, while B began to move away immediately (Figure 1A). For this sequence people typically report seeing A launch B, i.e., physical causality, confirmed by many independent studies (e.g., Schlottmann et al., 2006). A temporal delay of half a second or less at the point of contact destroys this impression in adults, as does a gap of a few millimeters. However, the overall impression depends on the configuration, affected also by speed and other factors, e.g., small gaps are tolerated at high speeds with small delays (Schlottmann and Anderson, 1993).

Later on, Kanizsa and Vicario (1968) also found perceived social causality in reaction events, very similar to launch events, but without contact (Figure 1B): a moves up to B, but B begins to move away before A could reach it. Both move simultaneously for a fraction of a second, then A stops and B moves away. Now people report social causality, that A is chasing B and B is running away, also confirmed independently (e.g., Schlottmann et al., 2006). Michotte and others, in particular White (e.g., White and Milne, 1997, 1999), have provided many other examples of what White calls interaction impressions, e.g., impressions of pulling, bursting, etc., typically for interactions in the physical domain, while work in the Heider and Simmel (1944) tradition suggests extensions to the social domain. In the present study, however, we focus on launch and reaction causality.

Perceptual causality independent of reasoning and learning, dependent only on particular structural features, as claimed since Michotte (1963), remains controversial for adults. After all, adults have relevant experience and a conceptual understanding of cause that they could bring to bear on interpretation of these simple motion sequences, which for the most part can be seen as representations of real causal events. Thus, White (2006) argues that causal interaction impressions arise when an event triggers a matching representation in memory, and the involvement of memory in adults' impression is difficult to rule out.

On the other hand, there have been multiple demonstrations of perceptual causality in infants (Leslie and Keeble, 1987; Oakes, 1994; Cohen and Amsel, 1998; Schlottmann and Surian, 1999; Schlottmann et al., 2009, 2012). Infants have little relevant experience and presumably lack an a priori understanding of cause that would allow them to identify particular sequences as potential cause and effect sequences. These demonstrations make a claim that causality is perceived more plausible, and many infancy researchers take this view (Leslie and Keeble, 1987; Schlottmann et al., 2009, 2012, see Scholl and Tremoulet, 2000).

In modification of Michotte's original claims, the view that causality is perceived need not imply that infants' and adults' experience does not contribute. Instead of a modular reading (Scholl and Tremoulet, 2000), the claim might be merely that there is a perceptual core to causal structure on which learning can build: if from infancy we see certain instances of causality even without relevant knowledge, this would support the acquisition of knowledge relevant to these causal events. Perception links the events together, and children can then figure out at their leisure why the events go together. These more rational and experience-dependent analyses of perceived causal links may well come to affect the perceptual impression subsequently. On this non-modular reading one can hold that causality is perceived, without denying that it is affected by experience and knowledge, thus sidestepping the controversy (Schlottmann, 2000; Schlottmann et al., 2009, 2012).

While perceptual causality has been much studied with adults and with infants, there is less work with talking-age children. Yet this is important—in infants perceptual causality can be inferred only indirectly from how long they look at launch and reaction events, but for direct evidence we need some form of perceptual report requiring language and therefore older children. The problem is, however, that children find it difficult to freely express their perception, and that the drawn-out, age-related changes found in early studies of children's verbal reports of launch and related events (Olum, 1956, 1958; Lesser, 1974, 1977; Thommen et al., 1998) could reflect development of perceptual causality or simply language development. That the latter is substantially involved follows from a study reporting perceived launch and reaction causality from age 3, when language requirements were reduced by asking children whether a picture of Postman Pat engaging in a physical or social or non-causal interaction fit various motion events (Schlottmann et al., 2002); the present study also adopts this picture methodology.

When we use a methodology other than free verbal reports, it is conceivable that observers may not report their spontaneous causal perception, but that the use of social and physical causal language is metaphorical, and that observers merely draw analogies between motion patterns on the screen and memories of real world events triggered, in this case, by stationary pictures or instructions. In the extreme, observers may not represent the causality of motions on the screen at all, but merely use such language after matching screen motion and event memory on lower level features. Adults, in contrast to this view, do spontaneously report causality in these types of screen events, and there is good agreement between results found with free report and structured responses, as typically used in contemporary studies (for review, see Scholl and Tremoulet, 2000; Schlottmann et al., 2006).

This worry is unfounded with children as well: it is already clear that even preverbal infants represent the causal, not just spatio-temporal structure of launch and reaction events, as reviewed above, and there is no reason to assume that this representation is lost in talking-age children. Moreover, that young children report analogies prompted by the instruction rather than spontaneous perception is even less likely than for adults: while pre-schoolers are capable of analogical inference, they are not prone to do so unless there is clear agent similarity between domains or knowledge of the underlying causal relations (see Goswami, 1992; Rattermann and Gentner, 1998; and discussion in Schlottmann et al., 2002). Note also that even the strongest Michottian claim is not that observers confuse these screen events with physical/social causation in the real world, it is merely that observers see one shape launching/chasing the other, being aware at the same time that these are all just shapes on a screen. Use of a structured methodology with children therefore would not seem to fundamentally change the nature of the task, but merely increases its sensitivity.

When such a structured picture methodology was used instead of free verbal report, pre-schoolers easily recognized basic forms of perceptual causality, but there were also age-related changes in the role of various perceptual cues (Schlottmann et al., 2002). From 3 years, children reliably identified launch events as instances of physical causality and reaction events as social causality, distinguishing these from non-causal events with a delay, but only 5-year-olds were as accurate in identifying non-causal events as they were in identifying causal events. Younger children often over-attributed causality, in part due to a causal response bias. This study thus suggests, firstly, that children's facility with the causal-noncausal distinction develops over the preschool range, while, secondly, the domain distinction seems well-established by age 3.

On the first point, it may seem surprising that children have difficulty with delayed events, given that infants perceive causality in causal but not delayed sequences. However, this makes sense when we consider that causal perception of some events does not imply non-causal perception of others. While launch and reaction events may be perceptually “special” with a relatively automatic meaning, this does not hold for delay events, which are perceptually neutral, without meaning. Children have to think about their interpretation, which is more difficult at younger ages.

The second point, that even the youngest children had no difficulty at all distinguishing domains of perceptual causality, agrees well with the standard position that from infancy interactions in the physical and social worlds are distinguished by whether the agents make contact or not (Premack, 1990; Mandler, 1992, 2004; Baron-Cohen, 1994; Leslie, 1994, 1995; Baron-Cohen and Ring, 1995; Carey, 2009): this is because mechanical interactions require spatial contiguity while social agents can also interact from afar. The absence of contact indicates that an action was self-initiated, and only social agents are capable of this. Concern with contact thus ultimately reflects concern with domain-specific mechanisms of causation.

For adults, of course, spatial contiguity is not the only cue to the causal domain. In perceptual causality, for instance, adults attend to animacy cues as well, attributing social causality more when the shapes move in apparently animate manner (Schlottmann et al., 2006), but this did not affect children, even though they recognized the movement as animate (Schlottmann et al., 2002). On face value, these data thus suggest late developmental change in how domains of perceptual causality are distinguished, with children, like adults, attending to contact relations from very early on, while other cues are attended only much later. The present studies evaluate this view.

Our first experiment reconsiders the previously found neglect of animacy cues: is this a true developmental difference, or could it be merely a secondary consequence of children's difficulties with temporal delays, discussed above? The inclusion of delay events in perceptual causality tasks may tax children's processing resources, and as a result children may not be able to attend to all cues available. On this view, if the task is simplified, by elimination of difficult non-causal events, this may free resources to attend to animacy. But this should continue to be neglected if there is a developmental difference in how children and adults distinguish domains of perceptual causality.

Should children be able to consider other than spatial information in Experiment 1, this raises the question whether the already established distinction of reaction from launch causality is best described as reflecting attention to contact relations (Premack, 1990; Mandler, 1992, 2004; Baron-Cohen, 1994; Leslie, 1994, 1995; Baron-Cohen and Ring, 1995; Carey, 2009), or whether temporal information plays a role as well. The issue arises because launch and reaction events differ spatially, but also temporally: one has contiguous, the other simultaneous motion. Thus, both types of information could underlie the earliest domain distinction in perceptual causality. Difficulty grasping the causal implications of temporal delays, described above, need not imply difficulty grasping the implications of temporal information more generally. Accordingly, Experiment 2 studies children's causal impression for displays varying temporal and spatial information independently, to assess whether temporal cues contribute to it as well. In this study, therefore, we move from considering temporal information as a processing factor that can impede or facilitate processing, to considering the cues to causality that it might provide.

Two further experiments consider at what level spatio-temporal cues might affect children's causal impression, in particular, whether children use these cues for inferences about the mechanism of causation. If children mainly consider whether a motion is self- or other initiated, as under the standard proposal, then they may treat reaction events with occluded motion onset as less social than standard reactions, inferring the possibility that contact might have occurred out of sight in the former (Experiment 3). Similarly, if displays have both simultaneous motion-at-a-distance and contiguous contact motion (Experiment 4), children may treat motion-at-a-distance preceding contact as more social than contact motion preceding motion-at-a-distance, because the latter is not ultimately self-initiated. If, on the other hand, children's causal impressions are relatively automatic reaction to particular perceptual configurations, then they might treat the two motion orders or occluded/non-occluded motion onsets similarly.

In sum, our experiments revolve around the domain-distinction in perceptual causality. While the standard view emphasizes the importance of spatial cues for a distinction between physical and social events, we consider in two experiments whether temporal and animacy cues may play a role as well. The standard view also holds that children use the perceptual information to determine whether motion-onset was self- or other initiated, and this is assessed in two further experiments.

We report the data as 4 experiments. However, one group of children participated in Experiment 1, while another group saw events relevant to Experiments 2 to 4, which are separated for convenience of analysis and argument. A third group of children provided additional data in Experiment 3 and 4, as noted below. Thus, we have four conceptually, but not always materially different studies.

## Experiment 1

The first experiment considers whether children can use other than spatial information in determining domains of perceptual causality. As discussed, previous work highlights children's difficulty with identifying non-causal delayed events. Here we consider whether this difficulty might affect children more widely: if it is processing-intensive to focus on whether events are non-causal, this may reduce ability to process other task components relying on the same resources. Elimination of the need to attend to non-causality may thus allow children to consider cues previously neglected.

**Figure 2:**
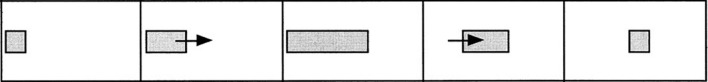
**Michotte's (1963) caterpillar stimulus**. The square appears to move itself by first rhythmically expanding from the right edge, then contracting from the left edge.

We assess, in particular, if with reduced processing requirements, children's causal impressions are affected by motion-style cues to the causal domain. Michotte (1963) first reported that a non-rigidly moving shape that rhythmically expands and contracts gives a strong impression of animate motion (Figure 2), and this appears for adults (Schlottmann et al., 2006), children (Schlottmann et al., 2002), and infants (Schlottmann and Ray, 2010). This animate motion also strongly affects causal impressions in adults (Schlottmann et al., 2006), but not children or infants (Schlottmann et al., 2002, 2009, 2012). Here we test whether this reflects processing limitations or a true developmental difference in perceptual causality.

### Materials and methods

#### Subjects

Sixteen 3-, 4-, 5-, and 7-year-olds (mean ages 3 years 7 months, 4 years 4 months, 5 years 3 months, 7 years 6 months; ranges 3 years 2 months to 3 years 11 months, 3 years 10 months to 4 years 9 months, 4 years 11 months to 5 years 10 months, and 6 years 9 months to 7 years 9 months), from London nursery and primary schools. Children consented orally and also had written consent from one parent.

#### Materials

Children chose from two A4 sized drawings to illustrate the target concepts. The same pictures were used as in Schlottmann et al. (2002), with the physical causality picture showing Postman Pat having kicked a football, while the social picture showed Pat chasing another man. Neither picture involved contact between Pat and ball/other agent.

Each child saw 4 different computer-animated motion events, made with Macromedia Director and shown on a Macintosh laptop attached to a 12 inch color monitor. Each event involved two squares (50 × 50 pixels, about 2.5 × 2.5 cm). Red (A) always started on the left, moved toward Green (B) in the middle of the screen and Green moved away toward the right. The motions repeated continuously, with about 0.6 s (30 frames) black screen separating cycles.

Two events were launch events, in which A moved toward B, contacted it mid-screen, and stopped, while B began to move as soon as A touched it. The other two were reaction events, in which A and B remained about 3 cm (60 pixels) apart. A moved alone for 30 frames, then A and B moved together for 30 frames, then A stopped, and B continued for another 30 frames. In one event of each type A and B moved rigidly at a rate of 4 pixels/frame (about 9.5 cm/s) over 60 frames. In the other event, the shapes moved non-rigidly, as in Figure 2. The square first extended over 10 frames at a rate of 8 pixels/frame with the left edge stationary, then it contracted at the same rate with the right edge stationary. After repeating these steps twice more, the non-rigid shape had covered the same 240 pixels distance in the same time as the rigidly moving shape. Each event took just under 5 s.

#### Procedure

Children were tested individually at their school. Children were first introduced to the response pictures and the target meanings were explained by questioning about their content. If children did not make appropriate statements, the experimenter (E) described the pictures to them as in Schlottmann et al. (2002). This was typically necessary for 3- and 4-year-olds.

Next children were shown the stationary squares on the screen. Children were told they would see different movies in which these squares would move, and to watch carefully so that they could explain afterwards what was happening in the movie; children were also told that the pictures would help them with this. Then the first movie was shown. After watching for an uninterrupted cycle, E pointed to the physical picture asking “does the green move because the red has hit, like in this picture?” She then pointed to the social picture asking “Or does the green move because it wants to run away from red, which is chasing it, like in this picture?” The event kept cycling until the child made a choice. Questions were typically not needed anymore after a couple of movies, with children pointing spontaneously, but questions were repeated as necessary. Sessions took about 10 min, with most of this time spent on initial discussion of the pictures. Movies were presented in individually randomized order.

#### Results

Table 1 gives the percentage of physical or social attributions to four events. The data replicate previous findings that all ages see contact events (rows 1 and 2) as largely involving physical causality, while non-contact events involve psychological causality (rows 3 and 4). However, in contrast to previous work, agent motion affected children's attributions: when contact events involved non-rigidly moving shapes they more often appeared as social causality, while non-contact events appeared more often as physical with rigid shapes (rows 2 and 3). The effect was strongest for the youngest children, who were split in their attributions.

**Table 1:**
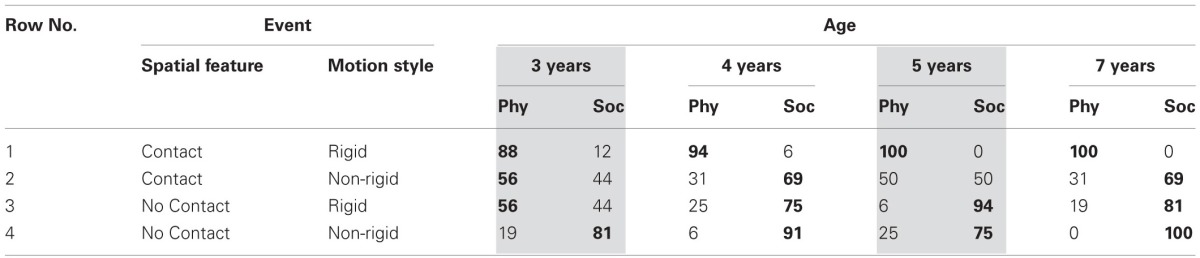
**Percentage of physically and socially causal attributions, in 4 age groups, for launch events with contact and reaction events without, each involving rigid, inanimate, or non-rigid, animate motion in Experiment 1**.

Modal values in bold.

Statistical analysis agreed with the visual impression. To enable factorial ANOVA, physical attributions received a score of 1, social attributions of −1. High proportions of physical attributions thus produce positive scores up to 1, while high proportions of negative attributions produce negative scores up to −1. Mixed responses move scores toward the chance level of 0. ANOVA effects can thus reflect the choice patterns in Table 1 (Lunney, 1970; Rosenthal and Rosnow, 1984; see Schlottmann et al., 2002).

The ANOVA here found not only a main effect of spatial information, *F*_(1, 60)_ = 101.76, *MSE* = 0.15, *p* < 0.01, η^2^_partial_ = 0.62, with more physical attributions to contact event, but also of motion style, *F*_(1, 60)_ = 42.21, *MSE* = 0.17, *p* < 0.01, η^2^_partial_ = 0.41, with more social attributions to non-rigid motion, and an interaction, *F*_(1, 60)_ = 22.26, *MSE* = 0.11, *p* < 0.01, η^2^_partial_ = 0.27, with non-rigid motion reducing physical attributions to contact events more than it increased social attributions to non-contact events. This asymmetry appeared for all but the 3-year-olds, leading to an age × motion style × spatial contiguity interaction, *F*_(3, 60)_ = 3.74, *MSE* = 0.11, *p* < 0.02, η^2^_partial_ = 0.15, and an age main effect, *F*_(3, 60)_ = 2.84, *MSE* = 0.13, *p* < 0.05, η^2^_partial_ = 0.12.

When the age groups were considered separately, all ages had spatial main effects, *F*_(1, 15)_ > 8.44, but, as described above, there was no interaction for 3-year-olds, with *F*_(1, 15)_ > 10.00 for the other ages. Five-year-olds had no motion style main effect, with *F*_(1, 15)_ > 8.44 for the other ages, due to a minor inversion in the data: at age 5, but not 3, 4, and 7, social impressions for non-contact events were slightly more frequent with rigid (94%) than non-rigid motion (75%). The reason is unclear, but the effect is small, with 5-year-olds, as all other ages, typically treating both non-contact events as social.

#### Discussion

This study for the first time found animacy effects on children's impressions of perceptual causality, with events involving non-rigidly moving shapes moving in an animal-like pattern generally producing more social, less physical responses from age 3. Overall, the pattern was similar to that found for adults, including that non-rigid motion affects launch causality more strongly than reaction causality (see Schlottmann et al., 2006). This asymmetry appeared for all but the youngest children.

The finding has two implications: first, there is not, after all, a developmental difference in domain-specific perceptual causality, such that this is affected by animacy only in adults. Rather, previously reported lack of animacy effects in children (Schlottmann et al., 2002) may reflect processing limitations. When the task requires thought about what delayed events mean, children may not have the resources to consider animacy cues at the same time, but when there are no delays, they do use animacy information.

Second, our finding highlights the different processing demands of spatial, temporal, and motion-style cues in perceptual causality tasks. An alternative view is that the animacy effects found here reflect more general changes in the task: they could be due to a reduction in response complexity, with two, not three response options. Or they might appear whenever children have to attend only to two cues rather than three. However, eliminating the need to attend to spatial information by presenting only contact or only non-contact events did not improve children's performance even though there were only two cues and two response options (Schlottmann et al., 2002; Experiment 3). This argues against these more general reasons for children's improvement here.

Instead, we propose that contact information is processed automatically in non-delayed motion events, even by young children, in contrast to delays and to motion-style cues, so there is little gain when the need to attend to spatial cues was omitted in Schlottmann et al. (2002; Experiment 3), but greater gain when delays were omitted here. This view fits with ceiling-level distinction of contact from non-contact causality from age 3, when children of the same age find it more difficult to distinguish causal from non-causal, delayed events (Schlottmann et al., 2002; Experiment 1 and 2), and when they attend to animacy only under simplified conditions, as studied here. Note that when both attention to delays and motion-style was possible (Schlottmann et al., 2002; Experiment 1 and 2), children attended to delays, not motion-style, even though one could argue that at a rational level the latter should be more important, with the non-rigid shapes appearing as self-propelled animate agents capable of social interaction, and there is no reason why such interactions should not contain delays. Nevertheless, delays seem more intrinsically important to perceptual causality than the nature of the agents.

In sum, the most important finding was that there is no developmental difference in perceptual causality between adults and children after all: all ages can consider animacy information in roughly similar manner. The experiment also further supports a view that some aspects of launch/reaction events are processed automatically, while others require attention, which may help explain why children are not always affected by all the same informers as adults.

## Experiment 2

If young children can attend to other than spatial cues in perceptual causality, then this raises the question whether their distinction of reaction from launch events really depends mainly on spatial information, as theories of infants early ontological distinctions suggest, following Premack (1990), or whether temporal information contributes as well, because launch and reaction events differ in both.

**Figure 3:**
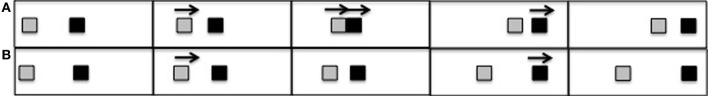
**Michotte's (1963) entraining event (A) and launching with a gap (B)**.

To study this, we varied temporal and spatial information independently. Each child saw 6 events, in which A and B moved simultaneously, contiguously or after a delay, either with or without contact. Contiguous contact events are standard launch events (Figure 1A), simultaneous non-contact events are standard reaction events (Figure 1B). Simultaneous contact events correspond to Michotte's entraining events (Figure 3A). Yela (1952) has previously described contiguous events with sizable gaps between the shapes (Figure 3B) as launching without collision, but Schlottmann et al. (2006) found that adults do not see such events as clearly physical. Gap events have not yet been studied with children.

As discussed, preschoolers have difficulty interpreting delay events. They do not lack perceptual sensitivity to the delay: infants as young as 2 months can detect delays of less than half a second (Lewkowicz, 1996), and 6-months-olds treat events with delays between 600 ms and 1 s as non-causal in habituation-of-looking-time studies (Leslie and Keeble, 1987; Oakes, 1994; Schlottmann et al., 2009, 2012). Nevertheless, 3-year-olds often ignore delays of that magnitude in causal attributions. Here, we increased the delay, to over 2 s, in an attempt to make it more salient to children.

### Materials and methods

#### Participants

Three child groups, 30 children each, participated, as well as 22 adults. The nursery group (18 girls) had children aged 3 and 4. The year 1 group (15 girls) had children aged 5 and 6, and the year 3 group (15 girls) had children aged 7 and 8. Children were from a London nursery and two primary schools. The adults (15 females) were typically undergraduates in their early twenties.

#### Materials

**Figure 4:**
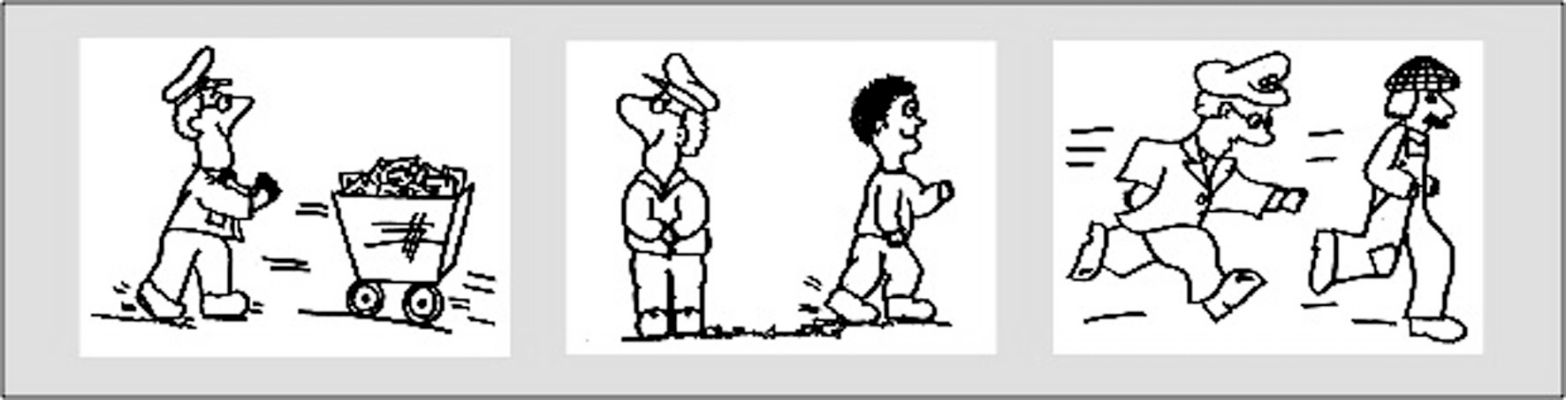
**Choice pictures for physical causality, non-causality, and social causality**.

Children chose from three A4 sized drawings to illustrate the target concepts (Figure 4), featuring Postman Pat pushing a post cart, Postman Pat standing while another walks by for non-causal, independent motion, and Postman Pat chasing someone who runs away. The agents did not make contact in any of these pictures. We switched the physical picture from the previously used football picture, to fit the entraining event better.

Events involved the same animated shapes, speeds and distances as before, but this time each child saw 6 different motion events, all involving rigid motion. In 3 events, A contacted B mid-screen, in the other 3 events, A and B remained about.6 cm (60 pixels) apart. In contiguous motion events, with and without contact, A moved first, and B began to move as soon as A contacted it. In simultaneous motion events, with and without contact, A moved alone for 30 frames, then both moved together for 30 frames, then A stopped, and B continued for 30 frames. In delayed motion events, A contacted B, and B began to move after 120 frames (about 2.5 s). The contiguous and simultaneous motion events took 240 frames in total (just under 5 s), with stationary periods at beginning and end adjusted; the delayed events took 260 frames.

#### Procedure

The procedure was as before. When the first movie was shown children were not just asked about the physical and social picture, but also about the non-causal picture: “Or does the green move on its own, not because of anything red has done, like in this picture?” Sessions took about 15 min, including the initial discussion. Movies were presented in individually randomized order.

#### Results

In Table 2, contact events without delay (rows 1 and 2) received about 90% attributions of physical causality, with no apparent age differences, and with no apparent difference between entraining and launch events. Thus, simultaneous motion *per se* is not a cue to social causality. Delayed contact events (row 3), in contrast, received far less causal attributions at all ages, and were typically seen as non-causal.

**Table 2:**
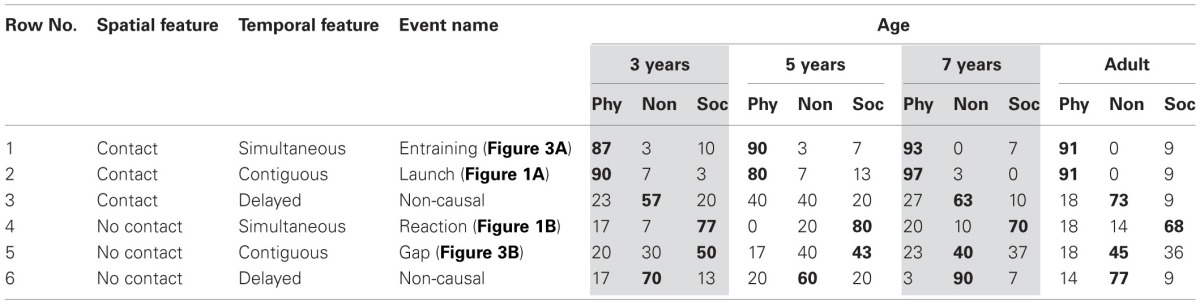
**Percentage of physically causal, non-causal, and socially causal attributions, in 4 age groups, for 6 events varying spatial and temporal features factorially in Experiment 2**.

Modal values in bold.

For no contact events, in contrast, it made a difference whether motion was contiguous or simultaneous: standard reaction events, with simultaneous motion (row 4), were treated as socially causal at all ages, while gap events with contiguous motion (row 5) received more non-causal, less social attributions. Delayed events without contact (row 6) were treated as non-causal, slightly more so than delayed contact events. Performance of the child groups on delay events was generally better than in previous work.

In line with the visual impressions, the ANOVA (as before, with score 0 for non-causal responses) found main effects of Spatial and Temporal Information, as well as an interaction, with the smallest *F*_(2, 216)_ = 8.53, *MSE* = 0.41, *p* < 0.01, η^2^_partial_ = 0.07. Follow-up tests showed no significant differences between attributions to simultaneous and contiguous contact events, *F* < 1, with high positive, physical scores for both. In contrast, simultaneous motion without contact received more social attributions, with more negative scores, than contiguous motion without contact, gap events, *F*_(1, 108)_ = 14.08, *MSE* = 0.56, *p* < 0.01, η^2^_partial_ = 0.11, which in turn had higher negative scores than delay motion without contact, *F*_(1, 108)_ = 6.85, *MSE* = 0.42, *p* = 0.01, η^2^_partial_ = 0.06. Delayed events with and without contact did not differ, with scores closer to 0, *F*_(1, 108)_ = 2.14, *MSE* = 0.31, *p* = 0.14. There were no age effects in any analysis, all *F* < 1.

#### Discussion

Experiment 2 shows clearly that temporal information is important for the domain distinction in perceived causality, not just spatial information. As in previous work, contact events were seen as physically causal, regardless of whether they involved contiguous launch or simultaneous entraining motion. Events without contact did not appear physical, and here the temporal structure mattered: while simultaneous motion without contact appeared as social causality, contiguous motion without contact appeared ambiguous, with more non-causal attributions. Physical reports of launching-at-a-distance appeared at no more than baseline level found for all stimuli, in contrast to Yela (1952). The same pattern appeared at all ages, including adult controls. It also replicates a recent study on adult free verbal report as well as ratings (Schlottmann et al., 2006). The difference to Yela's (1952) early results may reflect differences in stimuli, as well as instructions that allowed for both types of causality in the newer work.

The delay events here had 2+ rather than 1 s delay as in previous work. The extra long delay may have helped children, with even half of the 3-year-olds considering delayed events non-causal, an improvement over prior work, but as in previous work, children still had clearer impressions of causal than non-causal events. Age effects within this study did not reach significance, but non-causal attributions to delayed events still increased slightly with age, and appeared more frequently for delayed non-contact than contact events, also as in prior work.

Overall, Experiment 2 agreed with Experiment 1 that even young pre-schoolers attend to more than just contact relations when determining the domain of causality, in contrast to the standard view developed since Premack (1990). In particular, temporally overlapping, simultaneous motion is crucial for making non-contact events appear to show social causality.

## Experiment 3

Under the standard view, contact or its absence is so important for the domain distinction because this indicates whether the motion is self-initiated, and only agents are capable of this (e.g., Premack, 1990). On this reading, reaction events are seen as involving social causality, mainly because without contact, B is seen to self-initiate motion. The Experiment 2 finding that contiguous motion without contact does not appear as social causality is already at odds with this view. The next two experiments assess more directly the extent to which children's reaction to perceptual causality displays reflect concern with the onset of motion.

Experiment 3 assesses children's reactions to motions-at-a-distance with occluded onset. The logic here is that not all motions-at-a-distance are self-initiated, sometimes onset by contact may simply have occurred earlier, out of sight. Occlusion of the onset of motion thus allows for the possibility of earlier, unseen contact behind the occluder, and if the onset of motion is children's main concern, this manipulation should reduce impressions of social causality. On the other hand, if children ignore this distinction, treating all motions without contact, including occluded onset motions, as social, this would fit better with a view that children's impression is an automatic reaction to the perceptual configuration. A reduction in social impressions for occlusion events may, of course, grow with age, as children become more and more capable of integrating inference about the causes of motion with their perception.

**Figure 5:**
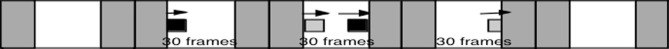
**Occluded reaction**. B (the black square) emerges already in motion from the left, followed by A (the lighter square); both then disappear on the right; the amount of visible motion at a distance is as in a standard reaction.

Children saw various motions-at-a distance with occluded onsets. In one event, both objects emerged, one after the other, already in motion from the left edge of the screen, and eventually disappeared behind the right edge. This event, compared to the standard reaction in Figure 1B, has a much longer period of simultaneous motion-at-a-distance, which in itself might make the event appear more social. Another event therefore had occluders to both sides of the screen, with B and A emerging in motion from the left and disappearing behind the right occluder, as if seen through a window in the screen (Figure 5). Occluders were spaced so that the event had the same amount of simultaneous motion-at-a-distance as the standard.

In two further occluder events, B moved faster than A, so that their distance increased over the course of their simultaneous motion. Backwards extrapolation then suggests contact some time before. B and A emerged once in such quick succession that contact was suggested just previously, behind the edge of the occluder. In the other event, contact was suggested behind the middle of the occluder. Although only simultaneous motion-at-a-distance was shown, inference about motion onset would in both cases not just allow for contact, but make this the likely possibility, which should reduce social impressions even further, if children are mainly concerned with motion onset.

The main issue, in sum, is whether events with occluded motion onset elicit weaker social impressions than standard reaction events, as the standard view would predict (Premack, 1990). Such reduction might be due to children making inferences about the possibility of contact motion. If this appears, it will also be important to show that this reduction is not simply due to the addition of occluders or reversal in motion order *per se*. To assess this, we had two control events. One had occluders placed in front of a standard reaction, so that B could still be seen to self-initiate motion. The other had no occluders and visible onsets, but B moved first, so that A ran after B, rather than chasing it away, as in the standard.

### Materials and methods

#### Materials

Events involved the same animated shapes as before, moving at standard speed from left to right, but the amount of visible motion differed. In edge-to-edge motion (row 1 of Table 3 in the results), B emerged, already in motion from the left edge of the screen, followed by A, both then moved across the whole screen, disappearing at the other side. This stimulus did not show motion onset, but 158 frames of simultaneous motion at a distance when the standard had only 30 frames. In the occluded reaction (row 2), therefore, black occluders were placed on the screen so that only 30 frames of simultaneous motion at a distance were visible, exactly as in the standard unoccluded reaction event. The event differed, however, in that B rather than moving from rest in the middle of the screen, emerged from the left occluder, and A disappeared behind the right occluder rather than stopping in the middle; also B moves first, rather than A. To test the effect of the occluders *per se*, we also had a control stimulus with a standard reaction: a emerged already in motion from behind the left occluder moving toward B in the middle of screen, which started from rest prior to A reaching it and disappeared behind the right occluder (row 5 of Table 3). These three stimuli had equal speeds for A and B, as in the standard.

**Table 3:**
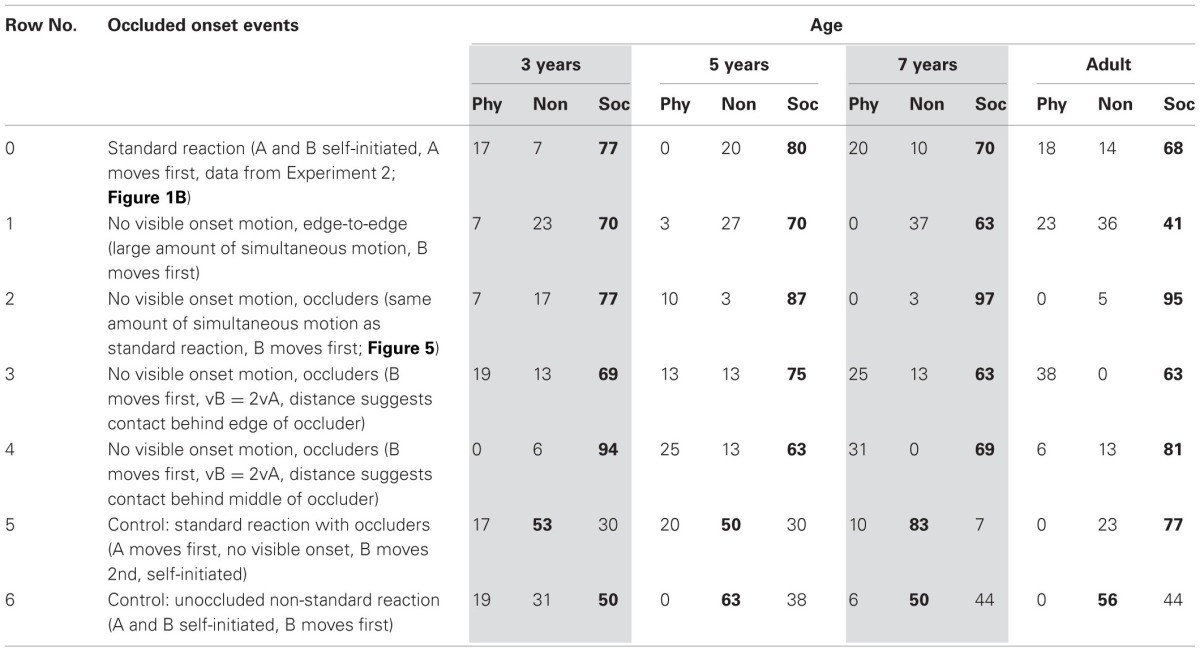
**Percentage of physically causal, non-causal, and socially causal attributions, in 4 age groups, for 4 events showing motion-at-a-distance without visible motion onset, and for 3 other events in Experiment 3**.

Modal values in bold.

In two further occluder events B moved at twice the speed of A, so over the course of movement their distance increased. In both events A and B moved simultaneously between occluders over 30 frames, but in one event A was only 8 pixels behind B when it first emerged, suggesting contact 2 frames earlier, just behind the right edge of the occluder (row 3). In the other event, A was 60 pixels behind B when it first emerged, suggesting contact 15 frames earlier (row 4). Finally, we had another control event, without occluders and with visible motion onset, but here B moved prior to A, rather than the reverse, as in the occluder events, but unlike the standard (row 6). Shapes had the same locations as in the standard, thus when B moved first, this increased the effective distance between the shapes during the simultaneous motion part to 300 pixels; it was only 60 pixels in the standard reaction when A moved first.

#### Subjects and procedure

The same children as in Experiment 2 participated, seeing the three stimuli with equal speeds (1, 2, and 5 in Table 4), and other stimuli reported later in Experiment 4, in a 10 min long second session on the afternoon of the day of Experiment 2. Children were briefly reminded of target concepts and questions, then the study proceeded as before.

**Table 4:**
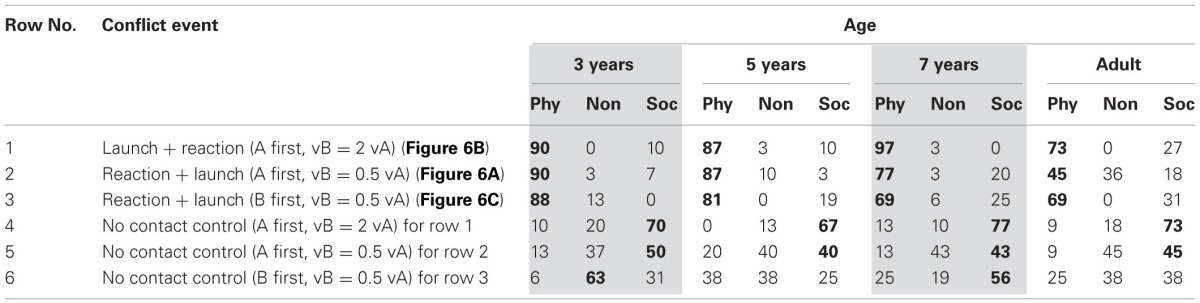
**Percentage of physically causal, non-causal, and socially causal attributions, in 4 age groups, for 3 events combining contact motion and motion-at-a-distance, and for 3 control events with the same temporal pattern, but without contact in Experiment 4**.

Modal values in bold.

As with any within subjects experiment, there is a possibility of carry-over and learning effects. In fact, we expect that children will indeed remember the general task set-up, in order to make instruction for the second session simpler. We presented the stimuli in two sessions to reduce noise in the data due to overly long sessions affecting children's concentration. The possibility of introducing artifacts, for instance, due to children communicating about the study in between sessions is slim, e.g., the verbal report studies (Olum, 1956, 1958; Lesser, 1974, 1977) show that children of this age cannot appropriately describe such stimuli. As for stimulus-specific learning effects, we randomized stimulus presentation within each session to control for this, but this does not, of course, preclude learning from the first to the second session. However, the only learning effects reported in the perceptual causality literature are stimulus adaptation effects within a given session for adults (e.g., Gruber et al., 1957; Powesland, 1959; Schlottmann et al., 2006; Woods et al., 2012), such that exposure to many causal stimuli increases sensitivity to and exclusion of stimuli with deviations from the causal category. It has, however, not been shown that such effects last beyond the experimental session. In any event, the first session stimuli here typically appeared half as causal, half as non-causal, so sizable adaptation effects would not be expected.

Another set of participants, 16 per age group, saw the other three stimuli (3, 4, and 6 in Table 4), also as part of a larger session, not reported here for the sake of brevity. The same procedure was used, but some stimuli were different. This set also involved children from different London nurseries and primary schools, and adults (mean ages 3 years 8 months, 5 years 9 months, 7 years 10 months, 40 years; range 3 years 3 months to 3 years 11 months, 5 years 1 months to 6 years 2 months, 7 years 0 months to 8 years 11 months, 19 years to 58 years).

#### Results

The first rows of Table 3 show that simultaneous motion at a distance elicits impressions of social causality even when motion onset is not visible and both shapes emerge already in motion. The impression is not at all reduced relative to the standard event (repeated as row 0) by the absence of self-initiated motion onset. Comparison of rows 1 and 2 shows that amount/duration of simultaneous motion does not matter: motion from one edge of the screen to the other (row 1) elicited the same proportion of socially causal impressions than when there were only 30 frames of simultaneous motion (row 2). Attempts to suggest the possibility of contact out of sight more strongly, by giving the shapes initial distances and speeds that imply contact behind the occluder (rows 3 and 4) produced at best small reductions in socially causal impressions. The single exception to predominantly social causality choices to occluded onset events is for adults seeing edge-to-edge motion (row 1).

These strongly social impressions are not due to incidental changes in event structure increasing social impressions and compensating for a reduction due to lack of self-initiated onset: the data in rows 5 and 6 show that the presence of occluders *per se* or a change in motion order if anything reduces rather than increases social impressions. The single exception here is again for adults whose social attributions are not reduced when the standard reaction is occluded (row 5).

Two ANOVAs were conducted, one for the stimuli shown to the first group of children (rows 1, 2, 5, as well as 0, the standard reaction data from Experiment 1), one for the stimuli shown to the second group of children (rows 3, 4, and 6), confirming the visual inspection. In the first group of children, a significant effect of event, *F*_(3, 324)_ = 18.88, *MSE* = 0.36, *p* < 0.01, η^2^_partial_ = 0.14, reflected the reduced social impression on the occluder control event 5, and an event × age interaction, *F*_(9, 324)_ = 4.84, *MSE* = 0.36, *p* < 0.01, η^2^_partial_ = 0.11, reflected that adults responded differently from children on this and the edge-to-edge motion event 1. In line with this, there were no significant effects of age or event, with uniformly high negative causal scores, when only the children's data were considered omitting event 5, *F*_(2, 174)_ < 2.47, *MSE* = 0.36, but adults differed from children on this event 5, as well as on the edge-to-edge motion event 1, *F*_(3, 108)_ > 3.09, *MSE* = 0.34, *p* < 0.01, η^2^_partial_ = 0.20.

When the three events shown to the second group of children were compared (rows 3, 4, 6), no significant effects appeared, *F*_(2, 120)_ < 1.69, *MSE* = 0.556, despite the reduction in social impressions apparent for the reversed-order control motion (row 6). This likely reflects lack of power with the smaller group size (*n* = 16 vs. *n* = 30), because a similar size reduction for the occluder control (row 5) was significant with the larger first group. However, this slight ambiguity in result for the reversed-order stimulus does not affect the overall interpretation. The reduction in control events with visible onset would have been important if events without visible onset had also shown a reduction relative to the standard reaction (row 0), but the events without onset showed no such reduction.

If the equal and different speed occluder events shown to the two groups of children are compared, then the small reduction in social attributions when contact behind the occluder is implied (row 2 vs. row 3 and row 2 vs. row 4) is significant in both cases, *F*_(1, 168)_ > 6.97, *MSE* = 0.328, *p* < 0.01, η^2^_partial_ = 0.04. The age × event interaction is significant as well in the comparison of event 2 and 4, *F*_(3, 168)_ > 3.99, *MSE* = 0.328, *p* < 0.01, η^2^_partial_ = 0.06, with 3-year-olds showing no such reduction in row 4.

#### Discussion

Events with simultaneous motion-at-a-distance but without visible onset are nevertheless seen as involving social causality, by adults and children. In the equal speed occluder event, there was no reduction in social impressions whatsoever, compared to previous experiments. When B moved faster and extrapolation from speeds and distances implied contact out of sight, there was a small reduction, mainly at the older ages, but even so responses remained predominantly social. If observers see motion-at-a-distance as social because it involves self-initiated motion, then occluding motion onset should reduce or eliminate social impressions, because one can infer a possibility of contact. This is not the case.

Substantially more motion-at-a-distance in the edge-to-edge stimuli neither increased nor decreased strength of the social impression for children, but for adults impressions were much reduced. One possibility is that adults expect contingent motions of animate agents not to be completely smooth and straight, but to show small variations in direction or speed. Our simultaneous motion, however, remained steady and constant over an extended period of time, which might suggest a mechanically rigid connection between the shapes. From this perspective, the event might be seen as a pulling event, as studied by White and Milne (1997). The children here, however, seemed oblivious to this possibility. Note that pulling is equally compatible with the equal speed occluder event (row 2 of Table 3), yet no age seemed to entertain that possibility.

While tangential to the main issue, it is also of interest why the control events showed a reduction in social impressions. In particular, addition of the occluder to the standard reaction (row 5), for children at least, eliminated social impressions even though the simultaneous motion was fully visible. The reason may be that the stationary B was initially visible mid-screen, while A's starting position was hidden behind the occluder. Thus, children may have focused initially on B which may have made them miss some of A's motion when it emerged to the left. This would not affect children in any other events, because all other events initially showed both A and B, or neither. Adults do not seem to have had a difficulty back tracking to A, even if only B was shown initially.

The negative effect of changing motion order (row 6) was less clear, not reaching significance, but this may be mainly a power issue. If we accept the reduction for the sake of discussion, then it might be attributed to increased distance between the shapes while moving. However, in an adult psychophysical study (Congiu et al., 2010), distance effects were minor. Moreover, in events 3 and 4 here distances ranged between 8 and 180 pixels, with no detrimental effects. A more likely account is that the changed motion order afforded a slight change in interpretation that did not fit our instructions well: in the standard reaction (Figure 1B), when A moves first, it chases B, and this in turn causes B to run away. Our instruction emphasized this view, but when B moves first from rest (row 6 of Table 3), B causes A to run after it. Both views are of action and reaction, and B moving first from behind the occluder fits equally with both, but event 6 does not quite fit the first interpretation. This mismatch may have reduced the social responses at all ages, in other words, we think this reduction, if reliable, is an artifact of the particular way social causality was instantiated here. Again, this is a side issue.

The most important finding here was that the occlusion events themselves showed little to no reduction in social causality compared to the data from previous experiments. Observers do not seem concerned with the onset of motion, but rather they react to the motion configuration *per se*.

## Experiment 4

As a second test of the view that children are mainly concerned with the onset of motion, we considered how children react to potential conflicts of features typical of social and physical events, i.e., involving both simultaneous motion-at-a-distance and contiguous contact motion, in sequences of either reaction + launch or launch + reaction events. In addition, this experiment also allows an assessment of the relative strength of children's reaction and launch percepts.

**Figure 6:**
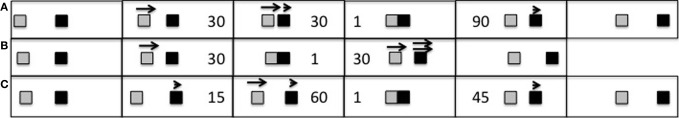
**Conflict events involving both contact and motion-at-a-distance**. The top **(A)** shows motion at a distance followed by contact when A catches up with a slower B. **(B)** middle shows contact followed by motion at increasing distance due to B moving at double speed. **(C)** bottom, again shows motion at a distance followed by contact with a slower B, but here, in contrast to **(A)**, the first shape to move is B. (Short and double arrows indicate halved and double speed relative to the standard; numbers indicate the duration in frames of each motion component).

To achieve reaction + launch and launch + reaction sequences, B moved either faster or slower than A. The reaction + launch sequence began as a reaction event, with A moving toward B, then A and B moved simultaneously. However, B moved slower, so A eventually caught up, made contact and stopped, while B moved on, as in a launch event (Figure 6A). Rationally, with concern for the onset of motion, this sequence should appear as social causality, because ultimately B's motion is self-initiated.

To achieve a launch + reaction event, in contrast, B moved faster than A (Figure 6B): initially A set B in motion, but after contact both continue to move simultaneously, as in a reaction, though at ever increasing distance. Rationally, this should appear as physical causality, because B does not self-initiate motion. A similar sequence might be observed in the real world in a collision of a much heavier A with a much lighter B.

A second reaction + launch event differed from the one already described in that B (rather than A) was the first to move, but A caught up and contacted B nevertheless (Figure 6C). We did this because we worried that if children do not show concern for the onset of motion in standard reactions or in the event of Figure 6A, this might be because they might initially be drawn to the first (A) motion, missing the onset of the second (B) motion which provides the crucial evidence for its self-initiated motion. If B moves first, this should draw more attention to the self-initiation of B's movement. Again, the self-initiated motion theory predicts more social responses for reaction + launch than for launch + reaction sequences.

What, in contrast, is expected if children react to the perceptual configuration *per se*, without inference about onset? Event order should not matter then, but, because all sequences contain both the social and physical configurations, they might appear ambiguous. Alternatively, one percept might be stronger and could dominate. For adults, launch is stronger than reaction causality (Schlottmann et al., 2006). For children, we do not know this yet; we only know they identify launch and reaction causality equally well. These conflict event sequences therefore also address if launch and reaction causality have similar strength. If so, conflict events might lead to ambiguous impressions. If, in contrast, one interpretation dominates, then this percept may be stronger.

Reaction + launch vs. launch + reaction sequences not only differ in causal order, but also in the shapes' speeds, so we needed controls for how these speed differences affected the impression. Accordingly, we also had identical motion configurations to Figures 6A–C, except that the shapes were further apart so never made contact.

### Materials and methods

#### Materials

Events involved the same animated shapes as before moving from left to right, and with A moving at the same speed reported previously, however, B moved either at half or double the speed, and the initial distances between A and B were adjusted as needed.

In the reaction + launch event of Figure 6A, B moved at half speed, so took 120 frames to cover the 240 pixels distance that A covered in 60 frames. First, A moved for 30 frames toward B, then both moved simultaneously for 30 frames with decreasing distance due to the slower B. After 60 frames of motion, A had caught up, made contact with B and stopped, while B continued for the remaining 90 frames. In the launch + reaction event of Figure 6B, B was twice as fast, so took only 30 frames to cover the distance, when A took 60. First, A moved for 30 frames, then contacted B, which began to move upon contact. Then both moved simultaneously at increasing distance for due to the faster B, and both stopped after 30 frames. In the second reaction + launch event of Figure 6C, B again moved at half speed, but this time B moved first in the reaction, not A. Initially, B moved alone for 15 frames, then both moved simultaneously with decreasing distance between them. After 60 frames of simultaneous motion, the shapes made contact, A stopped and B continued for another 45 frames. Corresponding animations without contact had identical temporal patterning, but the shapes were 60 pixels further apart initially, so never made contact.

#### Subjects and procedure

The same children participated as in Experiment 2 and 3. The first set of children saw launch + reaction and reaction + launch sequences of Figures 6A and B and corresponding non-contact control stimuli, interspersed in the same session as the occlusion stimuli of Experiment 3, in individually randomized presentation. The second set of children saw the second, non-standard reaction + launch stimulus in which B moved first (Figure 6C) and its control without contact, again as part of a larger session.

#### Results

Table 4 show that contact motion strongly dominates the impression: all conflict stimuli were seen as depicting physical causality, even if B self-initiated motion-at-a-distance before A made contact with it (row 2) and even if B was the very first shape to move in the sequence (row 3). Data for the control stimuli without contact (rows 4–6) show that the impression for these conflict stimuli is clearly not a function of the speed parameters used to create them, because these control stimuli elicit far more social impressions, in particular when B moves faster than A (row 4).

Adults, and possibly 7-year-olds, may give physical reports to conflict events slightly less often than younger children. This reduction was more pronounced when motion-at-a-distance preceded contact (row 1 vs. row 2), as expected under a rational evaluation that therefore B self-initiated motion. However, in the event of row 3, B was the very first shape to move, which should have made the self-initiated nature of B's motion even more salient, but for adults at least this did not produce less physical attributions (69%) than the launch + reaction sequence (73%). The data pattern here is not entirely clear.

Statistical analysis of the events given to the first group of children (1, 2, 4, and 5), found no difference between responses given to conflict events, whether contact preceded or followed motion at a distance (row 1 and 2), *F*_(1, 108)_ = 2.23, *MSE* = 0.36, *p* = 0.14, η^2^_partial_ = 0.02, in line with the main point that responses to both stimuli were uniformly physical. The age pattern was not clear in the statistical analysis either: the slightly reduced level of physical attribution at the older ages produced a small age main effect, *F*_(1, 108)_ = 4.98, *MSE* = 0.44, *p* < 0.01, η^2^_partial_ = 0.12, but this did not differ between the two events, *F*_(3, 108)_ = 1.95, *MSE* = 0.36, *p* = 0.13, η^2^_partial_ = 0.05, in contrast to the impression from Table 4. The patterns was not due to the speed differences: for the control events without contact (rows 4 and 5), there was no age difference; the only effect was that social attributions appeared more frequently the event of row 4, *F*_(1, 108)_ = 17.00, *MSE* = 0.46, *p* < 0.01, η^2^_partial_ = 0.14. When conflict and control events were compared, these differential patterns produced corresponding effects of contact, speed, an interaction, as well as an overall effect of age, the smallest of these effects, with *F*_(3, 108)_ = 2.98, *MSE* = 0.38, *p* = 0.04, η^2^_partial_ = 0.08.

When the reaction + launch stimulus shown to the second group of children (row 3) was compared to its no contact control (row 6), the only effect was an effect of contact, *F*_(1, 60)_ = 23.41, *MSE* = 0.71, *p* < 0.01, η^2^_partial_ = 0.28, again reflecting more physical attributions to the conflict event. When the two events showing motion at a distance preceding contact motion, but differing in whether A or B moved first (rows 2 and 3) were compared between the two groups of children, no difference appeared, *F* < 1.

#### Discussion

There are two main points to these results: first, children gave the same response to stimuli containing both contact motion and motion-at-a-distance, regardless of whether the event began as a launch in which B's motion appeared ultimately initiated by contact (Figure 6B), or as a reaction in which B ultimately self-initiated motion (Figures 6A,C). This result converges with the finding from Experiment 3 that children do not seem concerned with the onset of motion.

Second, the response children gave to these stimuli was strongly physical, even though the stimuli were ambiguous, involving a cue conflict. When the motion sequence includes an element of contact motion, this apparently dominates the impression. This was not a primacy or recency effect, because it appeared regardless of whether the launch element came first or last. This was also not a function of the differing speeds used to create the stimuli, because control stimuli with the same speed characteristics, but without contact at all, elicited more social than physical impressions, as expected. Adults may have somewhat weaker responses to the conflict stimuli, but the age effect was not entirely clear in the data pattern. Most importantly, however, adults, like children, typically had a physical impression.

## General discussion

Four experiments considered how children distinguish domains of perceptual causality in schematic motion events. The first two experiments showed that, contrary to prevailing opinion, even for pre-schoolers identification of physical and social causality does not only depend on spatial information. In Experiment 1, when temporal processing requirements were low, children's causal impressions were affected by whether or not the agents move like animals. In Experiment 2, a brief period of simultaneous motion was shown to be crucial for impressions of social causality in motion-at-a-distance events. Experiment 3 and 4 went on to show that simultaneous motion-at-a-distance is not important for social causality because it signals self-initiated motion: strong impressions of social causality arose even in occluder events that did not show self-initiated motion, but social impressions were eliminated in self-initiated motion events, if contact motion followed. Our results thus suggest that simultaneous motion-at-a-distance is an important cue for social causality independent of concern with motion onset, but also that contact motion is a stronger cue for physical causality.

Below we discuss the implications of these findings. First we evaluate the theory that children infer domain-specific motion onset from the perceptual configuration, then move on to the alternative view that domain-specific impressions are automatic reactions to specific perceptual configurations, including the issue of why contact causality appears stronger than social causality. Finally we consider the processing implications of temporal information in perceptual causality, including whether contact causality is processed faster than social causality, whether temporal delays between cause and effect affect perception in different ways than learning/inference, and whether different perceptual informers differ in processing demands.

### Are children mainly concerned with whether motion is self-initiated or not?

A classic view since Piaget (1969) holds that children attribute intentionality to objects that self-initiate movement without contact, which makes these object potential social agents. Premack (1990) updated this in an influential paper arguing strongly for parallel perceptions of causality and intentionality, such that from infancy self-movers are automatically seen as possessing agency, while (physical) causality is perceived when objects are propelled after contact. Gelman (1990; Gelman and Spelke, 1981; Gelman et al., 1995) further argued that humans of all ages are concerned with the causes of motion, with animates having internal sources of motion while inanimate motion is externally caused. In the modal view, therefore, contact motion signals from infancy that an event belongs to the physical domain, while motion without contact belongs to the social domain (see Mandler, 1992, 2004; Baron-Cohen, 1994; Leslie, 1994, 1995; Baron-Cohen and Ring, 1995; Carey, 2009). Typically this is discussed as rudimentary thinking about the causes of motion, with early inferences supported by innate biases to attend to perceptual correlates of each domain.

Importantly, under these views, contact or lack of contact is not important *per se*, but because it indicates externally vs. internally caused motion and thus potential for social agency. The debate as to where perception ends and thinking begins in infancy is perhaps unresolvable and certainly beyond this paper, but the prediction from this view would have to be that early perception/thinking should be consistent with rational expectations about motion onset, and that motion onset and other agency cues should be more important than spatial or temporal parameters *per se* in causal attributions.

In our study of domain-specific perceptual causality, however, neither children nor adults were concerned with whether the motion was self-initiated. Primary concern with motion onset would have predicted in Experiment 2 that contiguous motion-at-a-distance in gap events should appear socially causal, not just simultaneous motion-at- a-distance, and similarly we should have seen social attributions for conflict sequences in which motion-at-a-distance was self-initiated and preceded contact in Experiment 4. In Experiment 3, in contrast, we should have seen fewer social attributions when onset was occluded and contact out of sight possible/likely.

Our results were quite different. Despite self-initiated motion, gap events elicited at best ambiguous choices in Experiment 2, and reaction + launch events elicited physical choices in Experiment 4. Thus, self-initiated motion is not sufficient for perception of social causality. Self-initiated motion is not necessary for this either, because in Experiment 3, social attributions showed no sign of a reduction when motion onset was occluded. This pattern does not fit the view that domain-specific impressions depend on motion onset.

We should add that motion onset was not completely ignored: although social choices were less frequent in gap than reaction events, they were more frequent than in delay events, also slightly more frequent in simultaneous non-contact than contact motion in Experiment 2. When contact was strongly implied in the occluder Experiment 3, this led at least to a small reduction in social responses, and similar in Experiment 4 when self-initiated motion preceded contact, this possibly led to a small reduction in otherwise strongly physical responses, at least in older observers. But while concern with motion onset may have played a small role, it was clearly not observers main concern, at any age.

Our youngest subjects were 3-years-old, while the motion-onset theory was formulated with infants in mind (Premack, 1990; Mandler, 1992, 2004; Baron-Cohen, 1994; Leslie, 1994, 1995; Baron-Cohen and Ring, 1995; Carey, 2009), so it is possible that concern with self-initiated motion is predominant early on, but gets overridden with age. However, the theory is meant to align infant skills with later more rational expectations, and if older children and adults do not have these rational expectations for the present events, it undermines the theory, especially since we saw no developmental trends.

There is surprisingly little direct support for spatial information as perceptual basis for a domain distinction in infancy either. Demonstrations that infants perceive causality in launch events with and reaction events without contact are consistent with this view, but it is not clear at this point that preverbal infants even distinguish two domains of perceptual causality (Schlottmann et al., 2009, 2012). Infants' expectations of contact differ for animate agents and inert objects (Spelke et al., 1995), but this is the converse of the claim that contact/non-contact signals whether the action involves agents or inert objects. Only when self-motion is repeated and amplified, by second-long pauses and reversals of direction it appears to serve as an agency cue (Luo and Baillargeon, 2005; Luo, 2011).

Counter to the claim, on the other hand, are multiple findings that simple, non-repeated self-initiated motion is not sufficient or necessary for attributions of (social) causality or goal-directed agency in infants. (Gergely et al., 1995; Csibra et al., 1999; Schlottmann and Surian, 1999; Movellan and Watson, 2002; Shimizu and Johnson, 2004; Johnson et al., 2007; Schlottmann et al., 2009, 2012). This set of findings has not received much attention, because the data are usually from control conditions not of primary interest, but the studies converge on the idea that the simple contrast of contact/inert object and self-motion/animate agent typically assumed in standard theories of infants' initial ontology is too simple. Data from all ages thus seem to argue against Premack's (1990) claim that self-initiated motion is automatically perceived as intentional motion.

This is not to deny that infants differentiate self-initiated from externally caused motion, or that older observers can see such motion as intentional. Luo et al. (2009) showed that 5-months-olds had different expectations about the kind of activities a box they had seen to engage in self-initiated motion or not can undergo. They were not surprised if the self-moving box reversed direction, stayed still when hit, remained in mid-air when released, seemed to move behind an occluder. They were surprised when the same box, presented as inert, only propelled by a hand, engaged in the same actions. Infants were also surprised when either object appeared to vanish from behind an occluder or passed through an obstacle, so this not an expectation that self-propelled objects can do anything. Luo et al. (2009) argue that infants have a concept of self-propelled object as possessing internal energy. This enables a wider range of actions than seen in inert objects, but is not the same as that of an agent with potential to engage in social interaction. In Leslie's (1994, 1995) terms, one is a mechanical agent, the other an intentional agent. Self-initiated motion should be of primary concern as a cue to social causality only if it is directly linked to intentional agency. These views thus converge with the present position.

With older children as well, the results do not fit Premack's (1990) claim. It is easy for pre-schoolers to infer that animals move themselves (Gelman et al., 1994; Massey and Gelman, 1988), but there are age differences in the inverse ability to use self-movement as a cue to animacy/intentionality: Richards and Siegler (1986) showed that from age 7 children saw spontaneous motion as the most important cue to whether a novel object was alive, but younger children considered limbed motion instead. In Montgomery (1996), older 3-year-olds saw self-initiated motion of a human as more intentional than pushed motion, but younger 3 year-olds, even with extra help, did not do this to the same extent. Thus, there is little evidence that self-initiated motion is automatically interpreted as the motion of an animate agent, but humans clearly develop an inclination to make such inferences—when asked about animacy and when shapes differ only in that one self-initiates motion while the other is pushed, adults consider the former more animate (Gelman et al., 1995). However, animacy/intentionality does not come up in spontaneous descriptions of such stimuli, nor is self-motion always used to infer animacy when stronger cues are available (Schlottmann et al., 2006).

In line with this, we argue here that seeing self-initiated motion as intentional and thus potentially belonging to the social world is a possible interpretation, not a necessary perception, and also that observers do not consider this interpretation in domain-specific causal impressions of Michotte-type motion events.

### The social causality configuration: is minimally contingent motion-at-a-distance optimal?

If an inference of self-initiated or mechanically initiated motion onset is not crucial for perception of social or physical causality in children, then one alternative is that children's causal impressions are automatic reactions to particular perceptual configurations. In case of social causality, the effective spatio-temporal cue configuration may be the shapes' simultaneous, overlapping motion-at-a-distance. This cue is not just temporal, because simultaneous motion with contact appears entirely physical, as in the entraining events of Experiment 2, rather it is spatio-temporal, involving separated, but correlated motion paths, discussed also by Mandler (2004).

Does the reaction event provide the optimal instantiation of such correlated motion path configurations? Kanizsa and Vicario's (1968) reaction event was designed for a minimal contrast to launch events, allowing for demonstrations that minimal differences between events can lead to a switch in the perceived causal domain, so we know now that impressions of social causality do not appear only in events exceeding a certain level of complexity. However, in the conflict Experiment 4, the impression was not determined by the minimally correlated motion paths configuration but by the contact configuration. Moreover, in adults at least, social reaction impressions are weaker than those of physical launching (Schlottmann et al., 2006). This could be because the minimally contingent motion-at-a-distance used here is a passable, but suboptimal configuration.

One could speculate that stronger impressions of a social relation might be achieved with more extensive contingencies-at-a-distance, as in Heider and Simmel (1944), or Gao et al. (2009). Judging from adults' reaction to the edge-to-edge stimulus of Experiment 3, such extended contingencies should not be rigid and monotonous, but involve variations in paths, so as to not be mistaken for mechanical linkages, perhaps pulling with a rope or towbar between shapes (White and Milne, 1997).

However, the findings from children's perception of the edge-to-edge stimuli in Experiment 3 argue against this view: in contrast to adults, children did not have reduced impressions of social causality for these. This was the only sizable developmental difference in the present studies, which rather intriguingly suggests that impressions of a mechanical connection-at-a-distance might develop later, with experience. On the other hand, children did not have stronger social impressions either with the edge-to-edge stimuli than with more minimal path correlations. The present studies thus provide no clear evidence that extensive correlated motion provides a better cue for social causality than minimal motion-at-a-distance—and alternative views of asymmetries in performance as in Experiment 4 are discussed below.

### The physical causality configuration: faster to process or stronger than social causality?

Our studies also provided data on physical causality, confirming that the configuration for this includes both contiguous and simultaneous contact motion, launching and entraining (Michotte, 1963), so physical and social perceptual causality are not entirely parallel in this respect as well.

The main new finding here came from Experiment 4: physical contact causality dominated social causality-at-a-distance: observers reported physical causality regardless of whether the social configuration was also present, regardless of motion order, and regardless of concern with self-initiated motion. Contact causality thus seems to automatically draw attention and dictate the interpretation, while other aspects of the event are ignored. Strikingly, contact causality interfered with processing of preceding contingent motion-at-a-distance that would otherwise lead to a social interpretation. One might speculate that this ability of contact causality to override the usual reaction to a precedent contingent motion-at-a-distance depends on how temporally close the two perceptual configurations are. In the present study, contact occurred after 0.6–1.3 s of simultaneous motion. If this is insufficient time to complete processing, interference from the contact configuration might preclude that a social impression is ever achieved. Presenting more extensively correlated motion over a longer period may then well help boost the social interpretation of conflict stimuli, not necessarily because more extensively correlated motion is a better cue to social causality, but because it would provide extra time to complete processing. This account is not to deny the intrinsic advantage of the contact configuration: while launch causality interfered with preceding reaction causality, the reverse clearly did not appear, so either processing of launch causality is completed far more quickly, becoming resistant to interference earlier, or it is intrinsically stronger, as would appear from the adult data (Schlottmann et al., 2006).

A different reason previously considered for why social impressions tend to be weaker than physical impressions in adults was that even with similar strength perceptions, the schematic motion events are further from social reality involving real animate agents than from physical reality involving inert objects (Schlottmann et al., 2006). That imbalance in impression strength previously appeared only in adults, but not in children's choices, or infants' looking time data fit with the view that children are closer to merely perceiving the events, while adults interpret them. Our finding that all ages tend to see conflict events as physical does not fit this view, but the processing time account outlined above might help resolve the discrepancy. Further work on why asymmetries between physical and social causality occur is clearly necessary.

### Temporal delays in causal perception and causal inference

Temporal delay has received extensive attention, not just as a cue to (non)causality but also as a processing factor. In perceptual causality it is typically seen as a cue. Delays from 100 ms or so reduce the causal impression and by 200 ms or so it is replaced by the perception of two independent movements (e.g., Michotte, 1963; Kanizsa and Vicario, 1968; Schlottmann and Anderson, 1993). However, it is also clear that naïve observers seeing launch/reaction events for the first time tolerate far greater delays of a second or more (e.g., Michotte, 1963; Schlottmann et al., 2006). Formal studies of such adaptation effects (Gruber et al., 1957; Powesland, 1959; Woods et al., 2012) found that with more exposure to causal events, observers become sensitive to smaller delays. Short-term experience thus demonstrably affects causal perception. Such stimulus adaptation effects are common throughout perception, and can originate at the neural level (Helson, 1964; Clifford et al., 2007), posing little difficulty for a view that causality is perceived in a bottom–up way.

Children are generally more tolerant of delays than adults, frequently treating delayed launch and reaction events as causal when they rarely treat events without delay as non-causal. As argued earlier, this could reflect that delay events, in contrast to causal events, have no intrinsic meaning, so children need to make considered judgments, which improve slowly with age (Schlottmann et al., 2002). In infant looking time studies, no explicit judgments are required, and 6-month-olds have no difficulty separating causal from non-causal events based on the delay (Leslie and Keeble, 1987; Oakes, 1994; Schlottmann et al., 2009, 2012), but the shortest delay used with infants are about 600 ms. We can thus not entirely rule out that children have higher delay thresholds in causal perception, or that delays are less effective at degrading perceptual causality, perhaps as more long-term adaptation.

In the present study, in any event, children's non-causal responses improved relative to prior work, for events with extra-long 2+ s delays (Experiment 2). Performance was not quite at adult level, but the small age differences were not significant anymore. An alternative to this reflecting improved discriminability is that only at such longer delays it becomes noticeably more difficult to learn a causal relation, with the default response shifting from causal to non-causal.

To evaluate this possibility, consider that temporal contiguity effects appear not just in perceptual causality, but causal inference more widely, under conditions not conducive to causal perception, and that different processes could underlie contiguity effects in these other paradigms. For example, at a rational level, contiguity/delay effects can reflect concern with the time course of assumed causal mechanisms, for adults (Buehner and May, 2002, 2003, 2004) and children (Schlottmann, 1999). If a known mechanism requires a delayed effect, e.g., an energy-saving light bulb requires time to warm up (Buehner and May, 2004), or a ball has to reach a bell via a slow runway (Schlottmann, 1999), subjects choose delayed over contiguous causes. Thus, top–down effects mediated by pre-existing causal knowledge can reverse the usual cue relation between contiguity and causality.

Contiguity effects also appear in causal judgments of the link between subjects' own actions and their outcomes, when subjects may not be reasoning about mechanism, paralleling contiguity effects in instrumental learning in humans and animals (Shanks et al., 1989). In instrumental causal inferences, contiguous contingent sequences are judged more causal than non-contiguous contingent sequences, e.g., in Shanks et al. (1989, Experiment 3), 2 s delays reduced causal ratings slightly, 4 s delays reduced them more and as much as 8 s delays, but even then judgments remained well above those in a non-contingent control condition, so adults can learn causal links over extended delays. Occasionally, contiguity effects even appear in the absence of contingency (Anderson and Sheu, 1995, Experiment 4, delays between 250 and 8000 ms). We are not aware of any parametric studies of instrumental causal learning and contiguity effects in children, but for adults it would seem that contiguity effects in instrumental causality operate over a more extended time frame than in perceptual causality. This could simply reflect differential sensitivity of the tasks in disparate areas, but a domain difference appears even with identical tasks (Huber et al., 2004).

The implication is that while brief delays may disrupt perceptual causality and access to an automatic causal meaning for launch/reaction configurations, they may not yet disrupt a tendency to infer a causal link between events. This may only be reduced with much longer delays, and only then may young children begin to give reliably non-causal responses. In the gray zone of briefly delayed events, performance then depends on age, perhaps inhibition skills, knowledge, external scaffolding and other factors that might shift the response away from the causal default. This speculative account, separating the role of temporal information in causal perception from its role in causal inference, awaits further test, of course, and yet another possibility is discussed below.

### Do delay and motion-style information draw on common processing resources?

The above discussion implied that in causal inference as envisaged by instrumental theories, temporal information is not so much seen as providing cues toward causality, i.e., as information that points to/away from a causal relation, but as a processing factor. Rather than temporal information being represented explicitly, it constrains the computation, affecting the speed of learning/processing or the rates/probabilities of outcomes used to derive causal strength (see Buehner, 2005, for review). Another aspect of timing as a processing factor may be how it affects resource distribution. Causal inference (as opposed to causal perception) is typically seen as a domain-general process drawing on central resources, so if the inference is easier, more resources are left for other aspects of the task. If delay processing requires more resources than contiguity processing for children, this might then affect ability to consider further cues to causality. Such a resource account was considered here for Experiment 1.

In previous work, adults had shown strong effects of motion-style, with animate motion reducing physical impressions of launching, but enhancing social impressions of reactions, while children and pre-verbal infants seeing identical stimuli showed no effects (Schlottmann et al., 2002, 2009, 2012), despite all ages recognizing the motions as animate and inanimate. In Experiment 1 here, in contrast, children from age 3 showed clear reduction/enhancement effects as seen for adults previously, which we attribute to more available processing resources when the need to attend to non-causality was abolished. This result was crucial in showing that previously reported age differences in perceptual causality do not reflect a difference in perception, but merely task difficulty.

The need to share central resources may also help explain the improvement on delay events in Experiment 2 relative to prior work. Not only were delays longer than in previous work, as discussed above, but also the task did not involve non-rigid motion stimuli. Without need to attend to animacy information, children may have had more resources to cope with delays. Thus, Experiments 1 and 2 had complementary results.

To slightly modify the arguments from Experiment 1 in view of subsequent findings, the data all fit with the view of a processing hierarchy, such that non-delayed contact motion and simultaneous motion-at-a-distance are processed automatically and from early on, while events with alternative spatio-temporal configurations, e.g., involving temporal delays, or motion-at-distance without simultaneous motion, or events containing additional cues, e.g., about animate motion-style, require extra processing resources. Whether a task shows developmental differences then depends on the extent to which it draws on these more processing intensive elements. Michotte (1963) argued, for instance, that the shapes' speeds are also crucial for perceptual causality, but this has not been studied with children. It is controversial whether speed effects are perceptual or reflect rational physical inference (Sanborn et al., 2013), so developmental data showing whether speed is processed automatically and early, or whether, akin to animacy and delay, this requires resources and shows age effects, will be of much interest.

## Conclusions

These experiments clarify development of perceptual causality. Prior work showed that temporal information is important for distinguishing perceptually causal from non-causal events. Here we showed that temporal information also contributes to distinguishing domains of perceptual causality. First, contrary to prevailing belief, absence of contact is not the crucial cue for social reaction causality. The important cue is spatio-temporal in nature, correlated motion-at-a-distance. Equally contrary to prevailing belief, use of such perceptual information does not reflect concern with motion onset. Second, the temporal structure of the event is also important because it may affect ease of processing, as suggested by a trade-offs between attention to delay and motion style information, or by the dominance of physical causality in conflict sequences. We know little of the processes underlying perceptual causality, but consideration of the dual role of perceptual informers, as cues to causality and processing factors, might help move the debate beyond the long-ranging controversy on whether perceptual causality is modular or not (Scholl and Tremoulet, 2000; Schlottmann, 2000).

It is difficult to pin down in our study where causal perception ends and causal thinking begins. In our view, relatively pure causal perception might exist in young infants, but by the age children talk they have had much relevant causal experience affecting perception. Nevertheless, the absence of major age differences between 3-year-olds and adults, here and in previous work, shows that this experience does not slowly and gradually create a meaning for what before learning were meaningless artificial motions. Rather, in line with Michotte's views, launch and reaction events, even to young children and infants, have intrinsic causal meaning accessible from minimal information that experience merely modulates, not generates.

### Conflict of interest statement

The authors declare that the research was conducted in the absence of any commercial or financial relationships that could be construed as a potential conflict of interest.
